# Perspectives on common chronic diseases in adult cancer patients in South Africa

**DOI:** 10.1080/16549716.2023.2228567

**Published:** 2023-07-11

**Authors:** Maureen Joffe, Oluwatosin A. Ayeni, Witness Mapanga, Paul Ruff, Nivashini Murugan, Herbert Cubasch, Shane A. Norris

**Affiliations:** a Strengthening Oncology Services Research Unit, Faculty of Health Sciences, University of the Witwatersrand, Johannesburg, South Africa; b South African MRC and the University of the Witwatersrand Centre for Common Epithelial Cancers Research Centre (CECRC), Johannesburg, South Africa; c Department of Radiation Oncology, Faculty of Health Sciences, University of the Witwatersrand, Johannesburg, South Africa; d SAMRC/Wits Developmental Pathways for Health Research Unit, Department of Paediatrics, Faculty of Health Sciences, University of the Witwatersrand, Johannesburg, South Africa; e Division of Medical Oncology, University of Witwatersrand Faculty of Health Sciences, Johannesburg, South Africa; f Soweto Comprehensive Cancer Centre (SCCC), Chris Hani Baragwanath Academic Hospital, Johannesburg, South Africa; g Department of Surgery, Faculty of Health Sciences, University of the Witwatersrand, Johannesburg, South Africa; h School of Human Development and Health, University of Southampton, Southampton, UK

**Keywords:** Multimorbidity, noncommunicable diseases, cancer, low- and middle-income countries, South Africa

## Abstract

There is a rising noncommunicable disease (NCD) burden in low- and middle-income countries. Sub-Saharan Africa (SSA) bears a higher burden than the global average with South Africa (SA) enduring the highest regional burden. SA among other southern African countries also bears a high prevalence of HIV and other chronic communicable diseases. Having a perspective on common chronic diseases in the ever-increasing numbers of adult cancer patients in SA will inform our understanding of approaches to better manage them. This commentary reviews regional and national studies and data of low- and middle-income countries and particularly SA on the chronic infectious and NCD multimorbidity burden among adult cancer patients. It also reflects on the considerable health system challenges of managing discordant multimorbidity among adult cancer patients within the SA Public Health System. Despite the critical need to better manage the growing MM burden in general and particularly the high prevalence of discordant multimorbidity among cancer patients, there is a dearth of research into MM management generally and in LMICs particularly.

## The changing burden of disease trends is devastating low-and-middle- income-countries (LMICs) including South Africa

**Figure 1.**
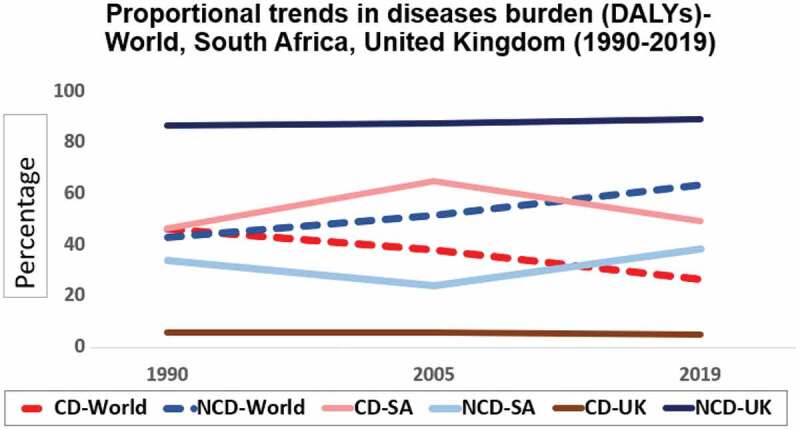
Proportional trends in diseases burden (DALYs)-World, South Africa, United Kingdom (1990–2019).

**Figure 2.**
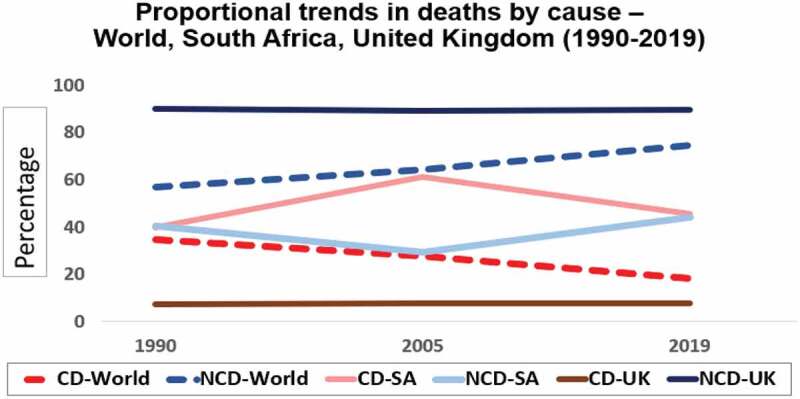
Proportional trends in deaths by cause-World, South Africa, United Kingdom (1990–2019).

Since the Second World War, medical advances have enabled substantial gains in the management of preventable and communicable diseases, resulting in steadily improving global health and increasing life expectancy. This infectious disease predominance is progressively being superseded by a rising noncommunicable disease (NCD) burden with the transition speed corresponding to the Human Development Index (HDI) of global nations [1–3]. This is dramatically illustrated in Figures 1 and 2, (data derived from the Global Burden of Disease Study and Our World in Data tables) [4,5]. By 1990, NCDs constituted the vast majority of the proportional disease burden in high-income countries (HICs), with LMICs beginning their transitions from 1990 onwards. In South Africa (SA), among other HIV endemic regions, the substantial HIV-related communicable, maternal, neonatal, and nutritional diseases (CDs) burden peaked in 2007, but with an effective antiretroviral (ARV) rollout these health burdens have been decreasing [6–9].

CD = Communicable, maternal, neonatal and nutritional diseases NCD = Noncommunicable diseases

Derived from Our World in Data tables (2019), https://ourworldindata.org/burden-of-disease

CD = Communicable, maternal, neonatal and nutritional diseases NCD = Noncommunicable diseases

Derived from Our World in Data tables (2019), https://ourworldindata.org/burden-of-disease

According to the World Health Organisation (WHO), NCDs kill 41 million people each year, equivalent to 74% of global deaths with over 80% of NCD deaths due to cardiovascular diseases (CVDs), cancers, chronic respiratory diseases and diabetes, and associated kidney disease [10]. Mental illness, especially depression, is the largest contributor to global disability (7.5% of all years lived with disability) with 80% of the disease burden in LMICs [11].

Some 86% of premature NCD deaths (age 30–69 years) occur in LMICs, Sub-Saharan Africa (SSA) bears a higher burden than the global average with SA enduring the highest regional burden [12–15]. Despite SA having the largest global HIV epidemic, its age-standardised death rates (ASM) from NCDs are now higher than those from HIV/AIDS and tuberculosis combined. CVDs are currently the leading category of NCD deaths in SA at 18.9% of all deaths in 2018 [7,16]. This is reflected in the fact that more than 40% of young people are inactive, 70% of women are overweight or obese, around 16% of the population smokes daily and ~18% engage in binge drinking [15]. Concomitantly, SA has the highest prevalence of hypertension (one in three adults) in the world, the diabetes prevalence among adults has tripled to around 13% since 2010 [17], and a large portion of hypertension and diabetes is undiagnosed [18,19]. The prevalence of chronic obstructive pulmonary disease is as high as 24.8% in some urban regions of SA and some 10% of the adult population experience clinical depression at some point in their lives with only around 25% accessing treatment [20].

GLOBOCAN 2020 reports that the global burden of cancer is substantially growing with the cumulative risk of developing cancer by age 75 at 22.6% for males and 18.6% for females [21]. In HICs, cancer has surpassed CVD as the leading cause of premature death^3^, but the largest percentage increases are now occurring in LMICs [22], and in SSA cancer is among the three leading causes of premature death (ages 30–69 years) [13,23]. In SA, the pathology-based National Cancer Registry (SANCR) provides data for cancer surveillance trends; however, as it is pathology-based, it under-reports the cancer burden, although regional population-based cancer registration has been published since 2017 [24]. Bray *et al.* derived their incidence estimates for the country based on death reports [13]. SANCR reported a cumulative lifetime risk (before the age of 74) for 2019 of being diagnosed with cancer in males at 16.0% (versus 23.6 from GLOBOCAN) and 12.4% for women (versus 18.7 from GLOBOCAN). In SA the highest incident cancers were estimated to be breast, cervix uteri, and colorectal cancers in females and prostate, lung, and colorectal cancers in males, with cancers with the highest age-standardised mortality rates (ASM) overall being lung, cervix uteri, breast, prostate, and oesophageal cancers [25]. Since the successful rollout of antiretroviral treatments in Southern Africa, the incidence of AIDS-defining illnesses, including AIDS causing (so-called AIDS-defining) malignancies, has been on the decline. Patients are ageing and are thus susceptible to NCDs including non-AIDS-causing (non-AIDS-defining) cancers.

## Multimorbidity burden

Unsurprisingly, the high chronic disease burden among global populations has created a perfect storm for multimorbidity (MM), defined as the coexistence in an individual of two or more NCDs (including mental health conditions) and infectious diseases of long duration. It typically occurs in ageing populations, but the global trend is towards a rapidly increasing prevalence at earlier age onset [26–28]. In HICs where the NCD burden is high, NCDs typically cluster together in varying combinations. Whereas in high CD endemic countries, clusters include chronic NCD and CD combinations which often act synergistically physiologically to exacerbate disease development and morbidity (e.g. HIV/ART which causes CVDs, tuberculosis, and diabetes which potentiate each other) [15,29]. Chronic diseases may cluster concordantly (e.g. cardiometabolic diseases such as hypertension and diabetes) where the comorbidities are related to the pathophysiology of the index disease and share the same treatment approaches [30]. They may also be discordant (e.g. cancer, mental health, hypertension, and HIV combinations) requiring separate management of distinct diseases, which poses enormous challenges to the current SA ‘single-disease model’ around which healthcare and research are traditionally organised [29].

## Evidence of discordant MM burden in South Africa

Our own evidence of the high discordant MM burden among SSA patients comes from breast and prostate cancer patients newly diagnosed and treated in tertiary academic public health hospital cancer clinics. Our studies span a single site (Chris Hani Baragwanath Academic Hospital, Soweto, Johannesburg), multi-SA (Johannesburg and Kwa-Zulu Natal) site, and a multi-SSA country site (SA, Namibia, Uganda, Zambia, and Nigeria) cohort study [31–39]. We confirmed a high chronic disease and MM burden among these low-, middle- and upper middle-income countries in young (aged 20–49 years) and older women (50–75+ years of age) with breast cancer (BC), with the higher HDI index countries (SA and Namibia) bearing the highest burden [38]. The MM burden among SA women was a high 44% and obesity (52.8%), self-reported hypertension (41.3%), HIV (22.0%) and diabetes (13.7%) were the chronic conditions that occurred most frequently [31,32]. The chronic hypertension and diabetes and overall MM burden among the male prostate cancer cohort,(average age 10 years older than the BC cohort) was also very high, though as expected the HIV prevalence among this group was lower (at 14%) [32]. Multimorbidity is characterised by frailty, functional decline and negatively impacts patients’ capacity to withstand, respond to, and adhere to cancer and other chronic treatments. Patients with MM tend to have poorer health outcomes, decreased quality of life, and greater mental health issues [40]. Our own results confirm this; we showed that HIV negatively impacts response to neoadjuvant chemotherapy, adherence to long-term tamoxifen treatments, and overall survival among BC patients and that multimorbidity burdens of two or more diseases co-existing with BC negatively impacted their survival [34–37,39].

## South Africa healthcare system challenges for multimorbidity management among adult cancer patients

The SA quadruple burden of disease has placed an enormous and increasing strain on the country’s under-resourced three-tiered public health system that serves approximately 85% of the population (which is largely socioeconomically disadvantaged and vulnerable), yet with only 20% of the available healthcare resources [41,42]. The impressive successes in integrating HIV and TB care have not been extended to the management of other chronic diseases [27]. The SA public health system is uncoordinated, and disease management is siloed, even at the primary healthcare (PHC) clinical level. This is particularly evident in the management of discordant MM among adult cancer patients. Cancer diagnosis and treatment are typically centralised in tertiary hospitals, already overburdened with the increasing cancer incidence, whereas other chronic diseases such as hypertension, HIV, uncomplicated diabetes, and mental health conditions are diagnosed and managed mainly in primary healthcare clinics, with only complex cases referred for tertiary management. Tertiary and PHC providers do not typically interact, and holistic, patient-centred MM management and shared decision-making are not prioritised. Consequently, cancer patients with MM must navigate polyclinics, general practitioners, and pharmacies in different healthcare facilities. Furthermore, many oncologists do not have clear MM management guidelines and backups to support them. This poses a significant risk for cancer patients’ overall quality of life and care [43].

The current consensus for best practices to manage MM is to focus on MM management at the PHC level, guided by the World Health Organization’s Innovative Care for Chronic Conditions (ICCC) framework [44]. It advocates that PHC providers need to be trained to be expert generalists to manage MM holistically through a tailored patient-centred care approach with skilled communication to cope with complex patient needs, circumstances and competing priorities, varying levels of health literacy and comorbidity of depression or anxiety [45]. The South African Department of Health responded in 2013 with a policy and strategy to re-engineer the PHC system to integrate chronic disease management into PHC services [46].

The United Kingdom National Institute for Health and Care Excellence (NICE) guidelines for MM management call for a re-orientation of care tailored to individual patients’ goals, priorities, and levels of health literacy [47,48]. It is appreciated that the inherent complexity of managing MM needs to be integrated into conceptual frameworks and guidelines that must consider the misalignment between patient needs and services provided [8,40]. There is evidence from work in SA that patients expected workload and capacity impact their healthcare utilisation and health outcomes [8]. Due consideration must also be given to the complex inherent biological interactions of multiple diseases, patient psychosocial, demographic and economic factors. Furthermore, their self-management capacity and capacity to meet health system expectations and demands of them, caregiver burdens and resources for support and quality of life impacts, must be considered. Provider knowledge and skills development in managing MM is required. In addition, health system capacity and re-organisation to manage MM require attention, considering existing community resources to augment scarce resources. Due consideration must be given to assessing patient frailty levels, considering all conditions and treatments simultaneously. Better data and guidelines about drug interactions and effectiveness of commonly prescribed treatments are required. We also require guidelines for stopping limited benefit treatments, introducing non-pharmaceutical treatments, and for coordinating follow-up visits and treatments. The development and recording of individualised management plans and the use of electronic records to facilitate communication between different providers dealing with patients are also fundamental requirements.

Despite the critical need to better manage the growing MM burden, there is a dearth of research into MM management generally and in LMICs particularly [27]. Recent reviews of interventions in HIC PHC settings have had very little impact on patient QoL and mental health [49,50]. Currently, no solutions exist, that we are aware of, that address the integrated management of cancer patients with chronic comorbid diseases. Going forward, we need to achieve many goals:

- More epidemiological evidence characterising cancer and MM burden in SA.
- Examination of existing policies and best buys, including expert health economic analyses, to determine changes needed to support the development, evaluation, and implementation of various interventions at the community, patient, and healthcare system levels to address the overall burden on the country.
- Provision of integrated preventative and disease management services, where the patient, as well as the community, are placed at the centre of care.
- Implementation of appropriate electronic tools and resources to enable ongoing surveillance of the burden of cancer and MM to determine trends and evaluate the effectiveness of interventions; and
- Activation of community resources and social structures and individuals and patients themselves to actively demand cancer and MM screening/early symptom detection services offered through preventative health services supported by the required policies. These goals are daunting but potentially achievable with effort and coordination across the stakeholder base.
- Involvement of all available public and private healthcare resources in so-called ‘private-public partnerships’ (PPPs) to enhance healthcare delivery.

## Paper context

In this commentary, we reported on the multimorbidity burden among patients with non-AIDS-defining cancers in South Africa. This is significant because the development and recording of individualised management plans and the use of electronic records to facilitate communication between different providers dealing with patients are fundamental requirements in multimorbidity management.
